# Non-Linear Interactions between Consumers and Flow Determine the Probability of Plant Community Dominance on Maine Rocky Shores

**DOI:** 10.1371/journal.pone.0067625

**Published:** 2013-08-05

**Authors:** Brian R. Silliman, Michael W. McCoy, Geoffrey C. Trussell, Caitlin M. Crain, Patrick J. Ewanchuk, Mark D. Bertness

**Affiliations:** 1 Department of Biology, University of Florida, Gainesville, Florida, United States of America; 2 Department of Biology, East Carolina University, Greenville, North Carolina, United States of America; 3 Marine Science Center, Northeastern University, Nahant, Massachusetts, United States of America; 4 Department of Ecology and Evolutionary Biology, Brown University, Providence, Rhode Island, United States of America; Michigan State University, United States of America

## Abstract

Although consumers can strongly influence community recovery from disturbance, few studies have explored the effects of consumer identity and density and how they may vary across abiotic gradients. On rocky shores in Maine, recent experiments suggest that recovery of plant- or animal- dominated community states is governed by rates of water movement and consumer pressure. To further elucidate the mechanisms of consumer control, we examined the species-specific and density-dependent effects of rocky shore consumers (crabs and snails) on community recovery under both high (mussel dominated) and low flow (plant dominated) conditions. By partitioning the direct impacts of predators (crabs) and grazers (snails) on community recovery across a flow gradient, we found that grazers, but not predators, are likely the primary agent of consumer control and that their impact is highly non-linear. Manipulating snail densities revealed that herbivorous and bull-dozing snails (*Littorina littorea*) alone can control recovery of high and low flow communities. After ∼1.5 years of recovery, snail density explained a significant amount of the variation in macroalgal coverage at low flow sites and also mussel recovery at high flow sites. These density-dependent grazer effects were were both non-linear and flow-dependent, with low abundance thresholds needed to suppress plant community recovery, and much higher levels needed to control mussel bed development. Our study suggests that consumer density and identity are key in regulating both plant and animal community recovery and that physical conditions can determine the functional forms of these consumer effects.

## Introduction

Understanding factors that regulate the recovery and secondary succession of communities following disturbances is a core focus of ecology and conservation [1]–[7]. In general, the species composition of plant and animal communities is thought to be driven by the combined effects of biotic interactions, the physcial charateristics of habitats, disturbance events and propagule supply rates [8]–[13]. For most systems, however, we know little about how propagule establishment is interacively controlled by resident consumer dynamics (e.g. density-dependence), trophic structure and local phsycial factors and how these interactions in turn determine community composition (e.g., biodiversity, spatial dominance, or the emergence of alernate community states).

The recruitment and establishment of plant and animal propagules in local communities can be under strong trophic control because consumers often create unoccupied space for new propagules to exploit (by consuming or disrupting competitors of the settlers) or by consuming or aggravating propagules after they have settled [14]–[20]. The strength of these top-down consumer effects is often a function of habitat type, consumer density and consumer species [21]–[23]. Although numerous studies have demonstrated that trophic structure can impact community development, we still have little appreciation for how the magnitude and direction of these consumer effects vary under different abiotic (i.e., temperature) charactersitics [21]–[23]. Indeed, field manipulations that addess the interactions between multiple biotic and abiotic factors are rare, in part, because of the complex and logistical challenge of such large experimental designs. Consequently, much of what we know about how the effects of comsumer density, identity and phsycial factors interact to impact plant communities has therefore been drawn from untested models [23].

In this study, we experimentally examined the combined effects of consumer assemblage and the physical factors that dictate propagule supply on recovery of macroalgae and invertebrates in a rocky intertidal community after disturbance. We found that consumer identity and density interact with abiotic processes (i.e. flow rate) to regulate recovery and that a keystone consumer can impose strong control over the composition and structure of communities that develop after disturbance.

## Methods

No specific permits were required for the described field studies, the experimental area is not privately owned or protected in any way, and no endangered or protected species were involved. This study was conducted on the Damariscotta River in central Maine. The Damariscotta River is a tidal estuary and its shores are lined with geomorphological features that create considerable variation in the strength of tidal currents over small spatial scales creating closely juxtopositioned habitat patches that expreince markedly different flow regimes. These differences in flow regime have been associated with consistent variation in the composition of benthic communities in high and low flow locations – spatially segregated areas of dense *Ascophyllum* (with Fucus interspered, especially in areas that have been recently disturbed) and mussel/barnacle beds (*Mytilus edulis* and *Semibalanus balanoides*, respectively) [24]. Habitats with low water flow (hereafter, low flow) are dominated by *Ascophyllum and to lesser extents by Fucus*, whereas habitats with high water flow (hereafter, high flow), often just a few meters away, are dominated by mussels and barnacles [24]. Chalk block deployment at 8 high flow and 8 low flow sites revelas that, on average, water flow rates at high flow sites are 3–4× greater [24].

### Trophic structure, flow, and the control of community reassembly

**Figure 4:**
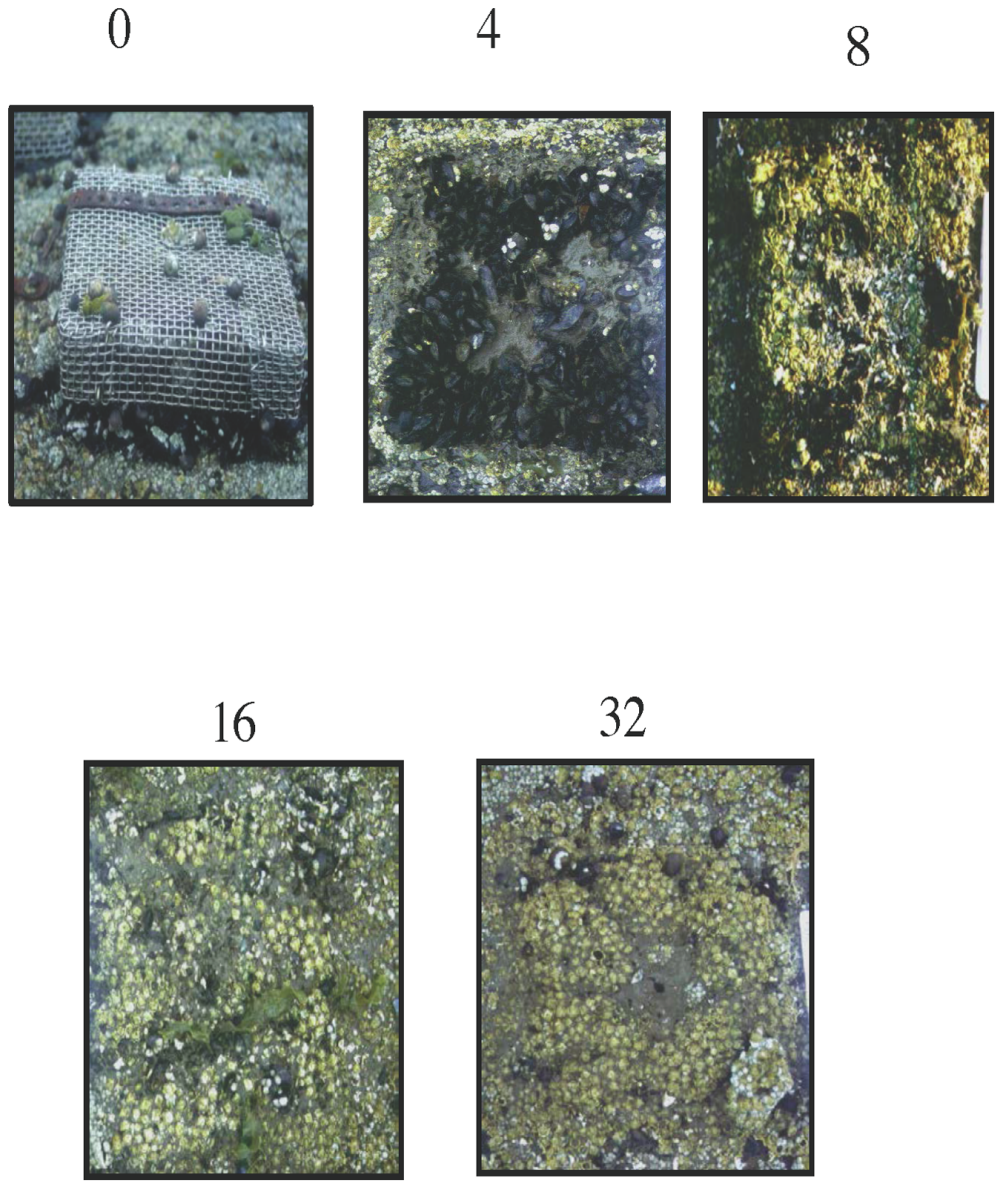
Pictures of representative impact of snail grazing at variable densities at high flow sites.

The two dominant invertebrate consumer species on the intertidal shorelines of this tidal river system are the green crab (*Carcinus meanus*) and the common periwinkle (*Littorina littorea)* [24]–[26]. The predatory snail, *Nucella lapillus*, and the northern yellow periwinkle, *Littorina obtusata* are abundant on the open coast of Maine but relatively rare in this tidal river (<0.5 individuals m^−2^) [25]. Therefore, we focused on the relative importance of periwinkle snails and green crabs in controlling the recovery of these communities following disturbance. At 5 high water flow sites (mussel bed dominated) and 5 low water flow sites (macroalgal canopy dominated) we created large >16 m^2^ (>4 m×4 m) clearings by removing all organisms from the substrate with flat edged shovels and hand scrapers [26]. These experimental sites were the exact sites used for the Bertness et al. 2002 [26]. In the Bertness et al 2002 study, replicated chalk blocks were deployed at the sites to compare relative dissolution rates and thus infer differences in flow. That data is presented in Figure 4 [26] and reveals that flow on average is 3–4 times higher at high flow sites. Since differences in flow does not vary much at all from year to year at the same site (its controlled by the morphology of the river) [24]–[26], we felt there was no need to redeploy chalk blocks for this study.

**Figure 1:**
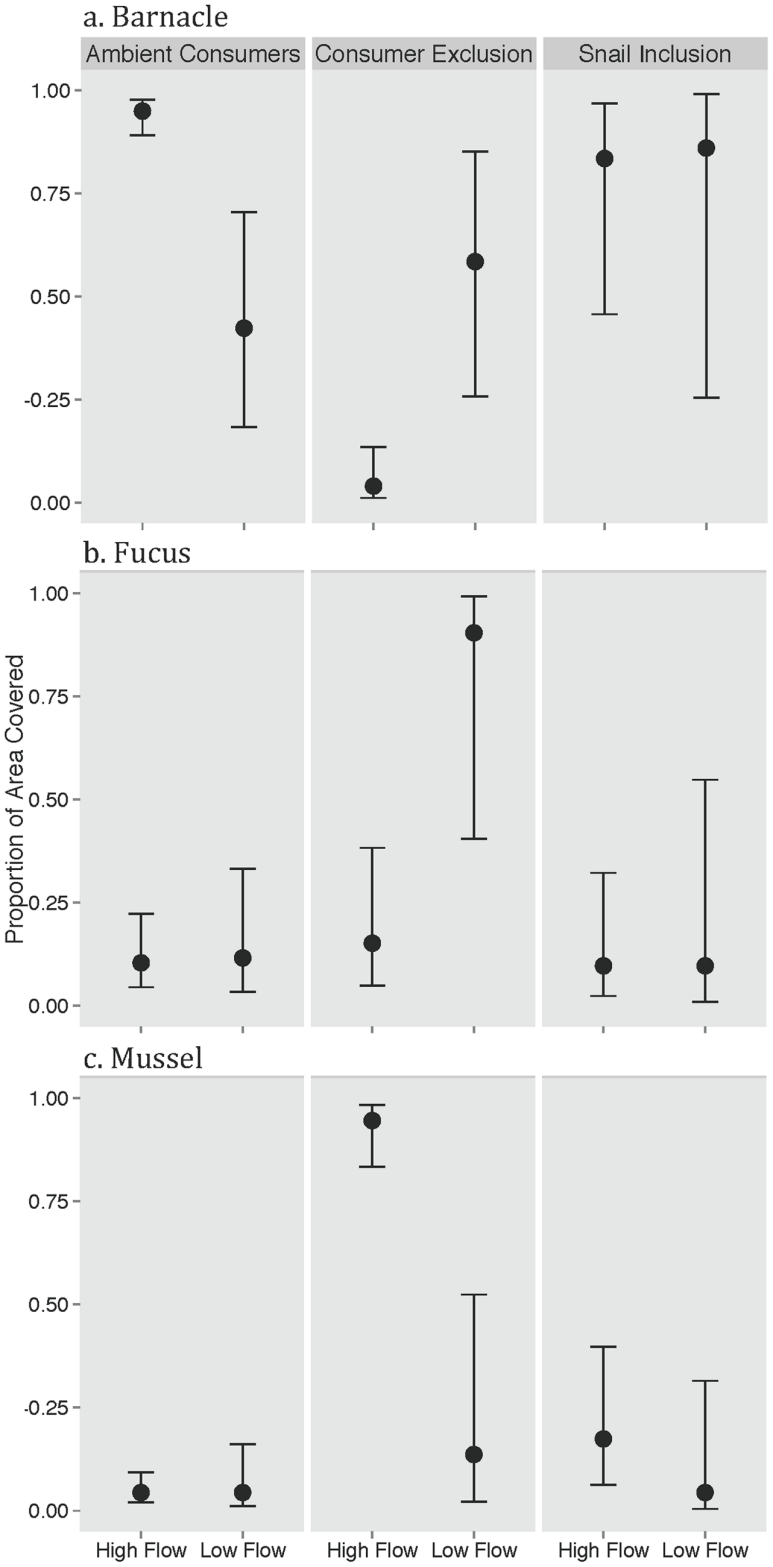
Species-specific (crabs and snails) consumer effects on on recovery of a. Barnacles, b. Fucus, and c. Mussels in experimentally generated bare patches at high and low water flow sites on the Damariscotta River. The data are presented as means ±95% CIs.

In each clearing, we marked and individually numbered 4, photographic monitoring quadrats (15 cm×15 cm). Quadrats were randomly placed in the cleared areas and marked by drilling corner holes and installing plastic anchors, screws and numbered plastic tags. All quadrats were burned with a propane torch to completely remove all organisms [26]. In each clearing, one quadrat was randomly assigned to one of four treatments (1) uncaged control, (2) caged-total consumer removal (i.e., snail and crab removal), (3) caged-crab only removal, and (4) procedural cage control. The total consumer removal quadrats were covered with a stainless steel cage (mesh opening: 5×5 mm; cage size 20×20×4 cm, L×W×H). The crab only removal cages were covered with identical cages, but snails (*Littorina littorea*) were included in the cage at ∼ ambient densities for each habitat type (see [26], Fig. 1 and Table 2; for *Ascophylum* sites n = 8 snails cage^−1^ ( = 108 snails m^−2^); for mussels sites n = 32 snails cage^−1^ ( = 512 snails m^−2^). Cages were not cleaned during the course of the experiment, as snails on the outside graze them and keep them clean of all visible fouling [24]–[26]. We used adult snails ranging from 22–26 mm in spire height and maintained average snail size so that it matched that found in our survey (see below, ∼ 24 mm). We used the most commonly occurring snail sizes (i.e., 22–26 mm in spire height – ∼65% of snails counted) to generate a mean size in the cages that matched that of the mean size out of cages as determined by our surveys. In this design, we infer the impacts of crabs by comparing snail inclusions to open plots (snails+crabs). This inference assumes that that effects of crabs + snails is additive. This non-interactive assumption seems reasonable given that green crabs do not typically eat large snails and that any non-consumptive effects of crabs should be equally present in all treaments given the small cage size. Cage control quadrats were covered with identical cages, but without sides. This experiment was set up in March 2001 and monitored photographically at the end of the experiment in September 2002. Snail densities were checked and maintained monthly (May-September) for the duration of the experiment. Importantly, our surveys of snail densities in uncaged control, caged control were not significantly different in mean *Littorina* abundance (P>0.24, one way ANOVA, for all months, both sites).

We tested for differences in the final percent coverage by barnacles, algae (*Fucus vesiculosus*), and mussels in high and low flow sites using generalized linear mixed models with beta distributed errors. For each analysis, habitat type (high and low flow site) and consumer treatment (i.e., control [i.e. crabs and snails], snails only, and consumer exclusion) were considered fixed effects, and each clearing location was considered a random effect. Analyses were conducted in the R statistical programing environment [27] using the glmmADMB package [28].

Intertidal densities of *Littorina* vary greatly in the Gulf of Maine, with stark differences in snail abundance occurring between both habitat type (high vs. low flow) and riverine versus coastal shores [25], [26]. To examine whether snail density affected recovery from disturbance (i.e., bare patches), we manipulated snail densities in bare patches at high flow and low flow sites using full cages identical to those described above and in the same 16 m^2^ clearings as described above. In each clearing, we marked and individually numbered an additional 11 photographic monitoring quadrats (15×15 cm) that were randomlly placed in the clearings. Quadrats were separated by at least 40cm, and substratum burned with propane torches. At each site, quadrats were randomly assigned to one of eight density treatments: 0 snails m^−2^, 16 snails m^−2^, 32 snails m^−2^, 48 snails m^−2^, 64 snails m^−2^, 128 snails m^−2^, 256 snails m^−2^, and 512 snails m^−2^. Field densities of adult snails (16–34 mm in spire height; mean  = 24.56+/−3.78) at the study sites was 437.3±87.5 snails m^−2^ at high flow sites and 92.5+23.6 snails m^−2^ at low flow sites. This experiment was also set up in March 2001 and monitored photographically in September 2002. During the summer (May-September) all snail density treatments were checked monthly and snails were replenished to maintain densities – this was rarely necessary. We analyzed these data with a mixed model beta regression using the glmmADMB package in R. Specifically, we tested whether the recovery (percent cover) by barnacles, mussels, or *Fucus* was a function of *Littorina* snail densities and flow rate. For this analysis, site type (high or low flow) was specified as a categorical fixed effect, snail density as a continous fixed effect, and individual clearings were again considered a random effect. All inferences are based on Likelihood Ratio tests and Wald's z tests.

## Results

### Trophic structure, flow and the control of community reassembly

We found a significant interaction between flow rate and consumers on the recruitment probabilities of barnacles (LRT, Χ^2^ = 40.51, df = 2, p<0.0001), *Fucus* (Χ^2^ = 17.98, df = 2, p = 0.0001), and mussels (Χ^2^ = 37.211, df = 2, p<0.0001).

#### Barnacles

In uncaged control plots, 95% of the area in high flow sites were colonized by barnacles when both snails and crabs were present (uncaged areas), but barnacle cover was reduced to 42% at low flow sites under the same consumer treatment (both consumers present) (Fig. 1a). In contrast, in the consumer exclusion plots, barnacle cover showed the opposite trend: barnacles covered less than 20% of the area at the high flow sites, and achieved nearly 60% coverage, on average, at low flow sites (Fig. 1a). In the presence of snails alone, however, baranacles covered more than 80% of the area, on average, in both high and low flow sites.

#### Fucus

Fucus coverage was low (<3%) in both high and low flow sites with both conusmers present in uncaged plots. However, in total consumer exclusion plots *Fucus,* domianated recovery producing a near monoculture (98.7% coverage on average) at low flow sites. However, *Fucus* never became established at high flow sites even in the absence of consumers (<5.5% coverage across all high flow treatments) (Fig. 1b). The absence of *Fucus* at high flow sites both in and out of cages likely occurs because there are few reproductive individuals in the area and *Fucus* is a local disperser [5], [24]. Fucus also failed to establish at low flow sites in the snail only treatements (3.2% coverage on average). These results suggest that snails alone are sufficient to strongly limit *Fucus* establishment at low flow sites.

#### Mussels

Consumers and flow rate also had a significant effect on the probability of mussel recruitment and establishment (Fig. 1c). In uncaged control plots (both consumers present), mussels were largely absent and covered <3.0% of the area on average at low flow sites and were slightly higher at high flow sites at <5% cover (Fig. 1c). In contrast, in complete consumer exclusion cages, mussels covered the entire surface at high flow sites and ∼ 15% at low flow sites. Mussels did not establish large populations in the snail only treatements at either high or low flow sites (∼1.5% of area covered on average). These results confirm those of previous studies in this system where strong consumer regulation prevented mussel bed recovery following disturbance [26] and indicate that snails (at these naturally occuring extremely high densities) alone can limit mussel recruitment [29].

### Snail Density Effects

**Figure 2:**
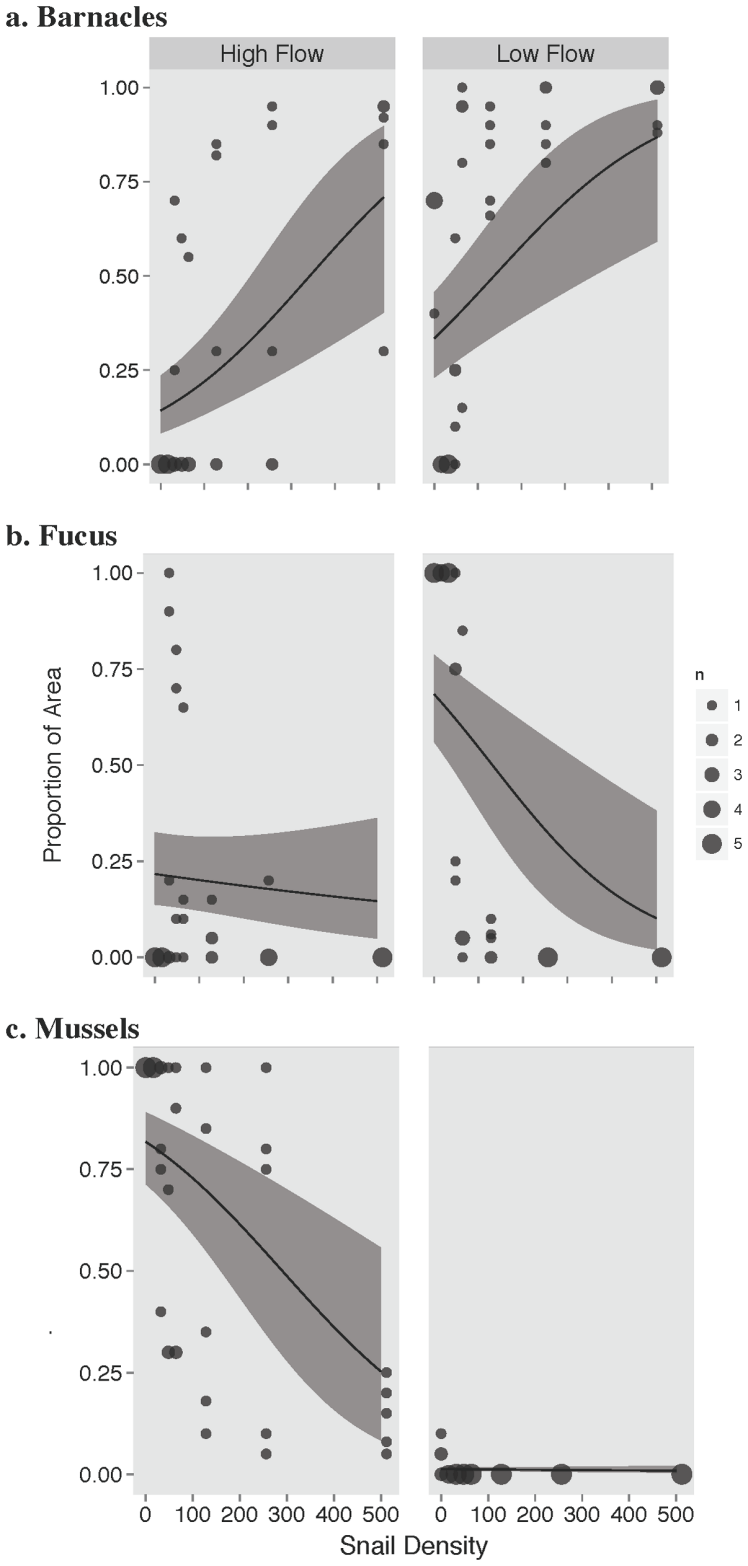
Effects of water flow rate and grazer (snail) density on a. Barnacles, b. Fucus, and c. Mussel recruitment in experimentally generated bare patches on the Damariscotta River, Maine USA. Symbol size depicts the number of data points occuring at that value, lines depict fits to the data using a beta regression, and shaded regions indicate the 95% confidence limits for the fitted line.

**Figure 3:**
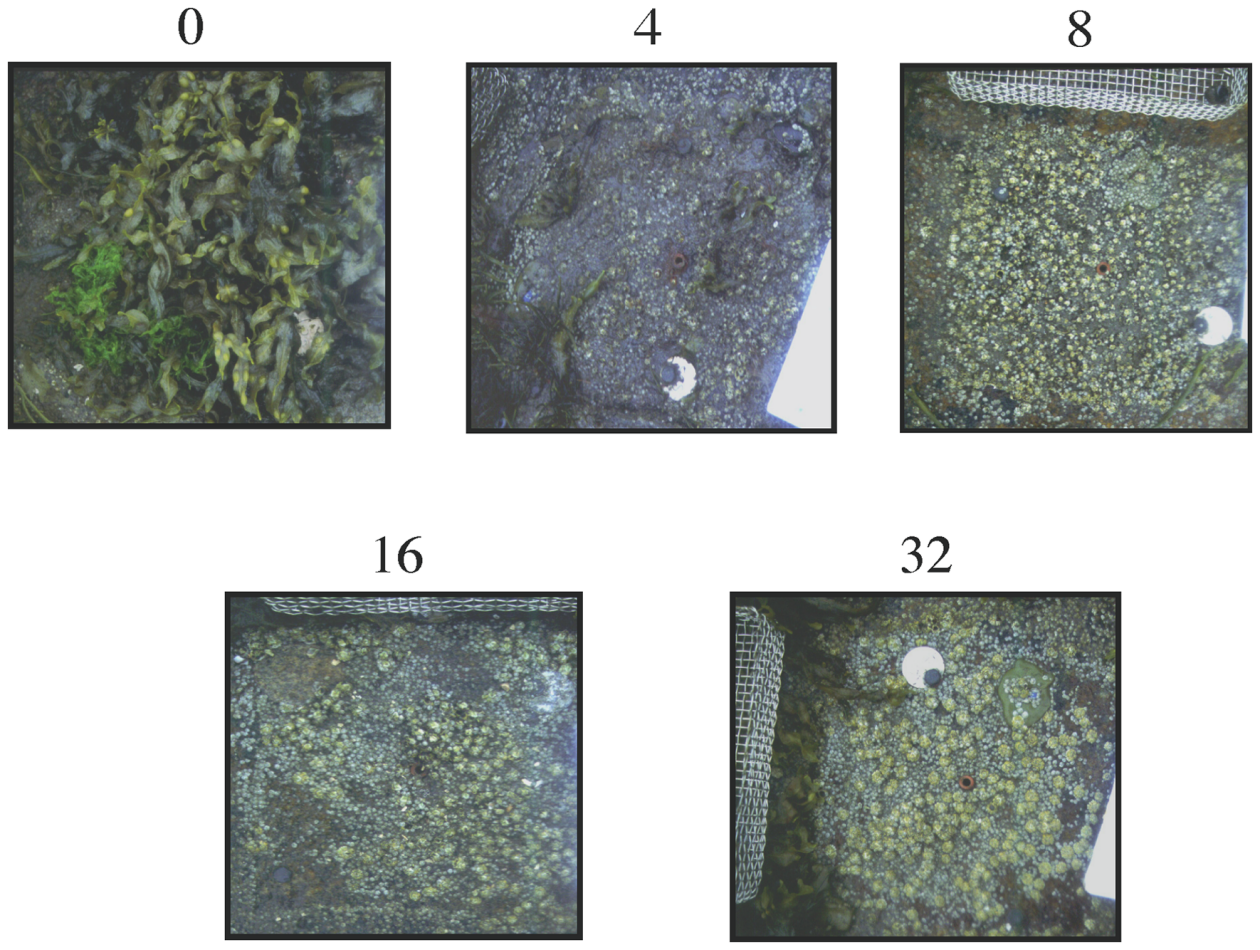
Pictures of representative impact of snail grazing at variable densities at low flow sites.

The interaction between flow rate and snail density significantly affected the probability of recruitment of *Fucus* (Χ^2^ = 10.164, df = 1, p = 0.001) and mussels (Χ^2^ = 12.57, df = 1, p = 0.0004), but not barnacles (LRT: Χ^2^ = 0.145, df = 1, p = 0.704) (Fig. 2–4). Coverage by barnacles was, however, affected significantly by the main effects of both flow rate (Wald's Z = 2.58, p = 0.01) and snail density (Wald's Z = 3.97, p<0.0001), with barnacles covering 42% more of the substrate in low flow sites but increasing in cover with increasing snail density in both low and high flow sites (Fig. 2a).

Fucus cover declined in both high and low flow sites with increasing snail density. Presence of low numbers of snails were able to regulate *Fucus* establishment at high flow sites – where Fucus recruits are less common, but higher densities of snails were required to regulate *Fucus* at low flow sites where recruits are more common (Fig. 2b). We observed a similar result for mussels except the highest density of snails in our desigin were needed to exclude mussels establishment, whereas snail density was relatively unimportant to mussel cover at low flow sites. Mussels were nearly absent across all snail densities in low flow habitats (Fig. 2c).

## Discussion

In many systems, stochastic settlement events are thought to be a dominant force regulating the assembly of plant and animal communities following a disturbance [25], [30]–[32]. However, the integral role consumers can play in driving the outcome of community assembly is receiving increased attention [33]–[36]. It is likely that both processes are playing important roles in most systems, but their relative contributions have often been difficult to disentangle partly because the biotic and environmental drivers of community recovery following disturbance have been confounded, thereby obfuscating pre- and post-colonization processes.

Most studies investigating consumer effects on community recovery have employed total consumer exclusions to isloate and quantify the net effects of consumers [25], [26], [37]–[41]. This method has been extremely effective in demonstrating the general importance of top-down consumer control to community organization [19], [42] and recovery after disturbance [26], [43], [44]. This experimental approach, however, does not discriminate the relative importance of propagule input rates, the effects of individual consumer species, or the role of density-dependent processes. A better understanding of the role played by variation in recruitment and species-specific and density-dependent consumer effects is critical for identifying key species and mechansims that are regulating community recovery [19], [45] and for predicting how natural- and anthropogenic-driven fluctuations in species' population densities will affect ecosystem structure and function [46].

Our results provide a unique demonstration that both consumer density and identity can be key regulators of whether plant or animal assembalges recover and dominate after a disturbance, and that both the shape and the magnitude of these density-depedent consumer effects are determined by abiotic conditions. Specifically, after ∼1.5 years, we found that: 1) plant and animal recovery from a disturbance in both low and high flow regimes on rocky intertidal shores in this tidal river are under strong consumer control, 2) grazing snails, more than predators, are the key bitoic agent imposing top-down control, and 3) that snail density and flow rate interact in non-linear ways to affect community composition.

At low flow sites, mussels were essentially excluded (likely by low larval delivery and bulldozing by low densities of snails), while the potential for *Fucus* to dominant these low flow sites (i.e., near 100% *Fucus* cover in all consumer exclusion cages) decreased dramatically and non-linearly with increasing snail density (Fig. 2). Only low to medium densities of snails were needed to generate the largest and disporprotionate suppression of *Fucus* establishement (Fig. 2 and 3). At high flow sites, mussels displayed contrastingly higher recruitment and dominated the rock surfaces unless snails were at their highest densities. Only at these highest densities were snails effective at suppressing mussel receuitment, and thus at the high end of the naturally-occuring density spectrum strong top-down control of community (i.e. mussels in this case) recovery can emerge. In these same high flow areas, *Fucus* did not show up or was extremely rare, likely reflecting the fact the *Fucus* is a local disperser and adults are not in these areas [47]. Barnacles, in comparison, were able to establish at both low- and high-flow sites, but in contrast to the pattern observed for *Fucus* and mussels, barnacle abundance increased with snail density. This positive association with snail density (Fig. 2a) likely occurrs because snails bulldoze sediment and dislodge settling mussels and *Fucus* from the surface leaving the space open for settlement by competitively-inferior barnacles [29], [44]. Another potential explanation for the positive assocaition of snails and barnacle cover is that snail suppression of *Fucus* removes algal inhibition of barnacle settllement that could occur through physical and/or chemical inhibition. Although snails can also negatively effect barnacle settlement, these inhibitory impacts appeared to have been overwhelmed by the positive effects of reducing sediment, mussels and algae.

### Consumer Identity- and Density-dependent Effects on Community Recovery

In this study, we show that in this marine-river ecosystem and during the time of the study predators (crabs) played a secondary role compared to grazers (snails) in controlling the recovery of disturbed rocky intertidal habitat patches. The most pronounced effects of having crabs in additon to snails on patch recovery occurred at low flow sites where crabs and snails limited barnacle abundance more than snails alone (Fig. 1), which is consistent with other studies showing that crabs can limit barnacle recruitment [48]. Green crabs do not commonly prey on adult barnacles, [48], but routinely consume recently settled, lightly calcified barnacle recruits. Crabs also slightly reduced the recruitment of mussels, but this effect was small in comparison to the impacts of grazing snails on mussel recruitment (Fig. 1 and 2). Although we know that crabs readily consume mussels [29], [44] their foraging efficiency may be depressed at the high flow sites because flows can disrupt prey localization (via chemical cues) and green crabs mobility [48].

Experimental manipulation of periwinkle snail abundance demonstrated that in high densities snails alone can influence the composition of the community that assembles after disturbance in both low and high flow habitats types. At low flow sites, snail grazing even at low densities of snails (48–128 snails m^−2^) suppressed percent cover by fucoids, cleared the substrate of sediment, and facilitated barnacle success (Fig. 2A). Moreover, green macroalgae (i.e., *Ulva* and *Entermorpha* spp.) were only found in cages without snails [29], [49], [50]. At moderate and high densities (256–512 snails m^−2^), snails entirely prevented algal establishment at low flow sites, even though adult barnacles were present and are known to facilitate fucoid establishment by increasing refugia from grazing [17]. In addition to limited larval supply at low flow sites, snail grazing also limited mussel establishment, likely through bulldozing and/or the elimination of dense algal canopy, which is known to attract mussel recruits [29], [49], [50]. The interaction between flow and consumers, where higher flow environments dampen top-down effects, has been observed before in this [26], [48] and other intertidal systems [13], [43], [51], [52]. Our study expands this knowledge by showing that these interactions are density-dependent and that increased supply of mussel recruits in high flow habitats likely preempts the consumer suppression of community development observed at low-flow sites. In other words, high mussel recruitment at high flow sites swamps out the suppressing influence of top-down effects.

We caution the extrapolation of our species-spefiic results to other similar rocky shore systems without additional experiments at those sites. Because predator diversity was low at our tidal river sites (primarily just *Littorina* and *Carcinus*) compared to more open coast areas where drilling snails, more crab species, seas stars and urchins occcur (e.g., 26) and because we could have conducted this experiment during years when green crabs were at realtively lower densities (we did not measure crab abundance but inferred relative densities based on past studies at these sites which did measure green crab abundance [24]–[26]), our results showing that snails are more important than crabs in controlling community development are likely to be spatially and temporally variable and depend on consumer diveristy, relative densities, and year of study.

### Implications for understanding alternative community states

Ecologists have long argued whether natural communities of plants and animals are deterministic products of specific environmental conditions or stochastic products of chance recruitment events [53], [54]. Recently, the debate over the deterministic nature of natural communities has shifted to discussions of whether assemblages of organisms can commonly occur as stochastically generated alternative stable community states [55]–[59]. These debates are not simply academic exercises, because understanding the relative importance of deterministic versus stochastic processes in community development has important implications for the conservation, management and the restoration of natural communities [60]. Our results concur with past studies [26] and show that secondary succession in low and high flow habitats on rocky shores in this Maine tidal estuary are likely the outcome of the combined effects of stochastic events (disturbances), environmental forcing (i.e. flow rate), and consumers. Our results reveal that consumer species and their densities set the context under which top-down control is expected and that the thresholds for these effects are regulated by the abiotic flow regime. Thus, understanding how the effects of species identity and density interact with environmental factors will likely be essential to make robust predictions regarding community recovery from natural- and anthropogenic-driven ecosystem disturbances and should be incorporated into future studies in this and other ecosystems [45], [46].
